# Incomplete and mismatching descriptors of scanning electron microscopy (SEM) and SEM microscopes: a case study

**DOI:** 10.17179/excli2025-8605

**Published:** 2025-08-08

**Authors:** Jaime A. Teixeira da Silva

**Affiliations:** 1 Independent researcher, Ikenobe 3011-2, Kagawa-ken, 761-0799, Japan

**Keywords:** ethics, honesty, medical communication, scientific ethos, SEM, TEM, truth

## Abstract

The post-publication scrutiny of the literature occasionally reveals errors that have filtered past the scrutiny of peer reviewers and editors. Microscopes, as used in scanning electron microscopy (SEM), form an integral part of the evidence-based methodology of many biomedical studies. A 2025 preprint (DOI: 10.31219/osf.io/4wqcr) claimed that a body of literature in indexed and ranked journals may have published potentially incorrect microscopy (SEM)-based evidence, noting that in about 2400 cases, the model or maker of SEM microscopes, as indicated in the text (e.g., in the methodology section), do not match information indicated in the figures or micrographs. One possible explanation may be that those analyses and/or equipment may have been outsourced to third-party services, although the outsourcing was not declared. Homing in on a sub-set of that preprint's 2400 cases, looking specifically at 23 of the 94 papers published in the mega open access journal, *Heliyon*, that were flagged in that exposé, textual descriptors in the methods section were compared against SEM descriptors in figures' micrographs. Only two papers showed an unequivocal discord between textual and figure descriptors related to SEM at the level of model and maker, while 16 of the 23 papers had no methodological description of SEM in the methods section. *Heliyon* editors need to investigate these omissions and discrepancies, and correct the articles accordingly, wherever applicable.

See also the graphical abstract(Fig. 1).

## SEM for Biomedical Research

The microscope is an instrument that allows a user to gain a finer-scale appreciation of a specimen sample than if they were to use their naked eye or a dissecting light microscope. When greater detail about the surface of a sample is needed, particularly at a three-dimensional scale, then a more granular analysis is required, as often occurs in biomedical research, this necessitates specialized equipment. Microscopes that need to analyze sample surfaces at the micro/nano scale, such as in scanning electron microscopy (SEM) (Varsano and Wolf, 2022[7]), are manufactured by specialized companies (e.g., Hitachi's SEM microscopes produced in Tokyo, Japan) because this technology is complex and expensive. Given the cost of such microscopes, not all research institutes have the budget to purchase their own SEM, and researchers who do not have equipment for SEM analyses might prepare samples in-house, then send them to an external company, or a third party service (TPS), to conduct the analyses. In those outsourced analyses, the results, including details of the SEM microscope and the micrographs themselves, are then returned to their clients, who would use them in scientific publications.

## Outsourcing SEM Analyses: Ethical Limitations Exposed by Post-Publication Peer Review

The use of any TPS - in this case, sample outsourcing for SEM analyses - must be declared by authors when they submit papers to journals that claim to follow standard or established ethical rules, even more so in the biomedical sciences, and failure to disclose the use of any TPS is a *de facto* ethical infraction (Teixeira da Silva et al., 2024[5]). During post-publication scrutiny of the biomedical literature, errors that have filtered past peer reviewers' and editors' scrutiny can occasionally be identified and revealed (Yeo-The and Tang, 2023[8]). Depending on the level of error, such papers may be subjected to a simple erratum, but if such papers carry serious errors, or evidence of fraud or fakery, such as the failure to disclosure the use of a TPS, including services that conducted microscopic analyses (e.g., SEM) on behalf of the authors, then such papers may be subjected to retraction, following an ethical investigation by the journal or their research institute (Teixeira da Silva, 2022[4]). This may or may not be the case for the papers described next.

## Are SEM Descriptors in Indexed Literature Discordant? The Heliyon Case

In a preprint-based *exposé* related to the potential misidentification of SEM microscopes (Richardson et al., 2025[3]), 11,314 papers were screening from indexed and metrically ranked journals, and about 2400 were flagged, with the claim that information in the text (e.g., methods) did not match the SEM descriptor in the figures' micrographs. This paper focused on one open access mega journal (OAMJ), *Heliyon*, published by Cell Press (Elsevier), with potential indexing at Web of Science being placed on hold in 2024 due to issues with quality control (Joelving, 2024[2]). Concerns have been raised about scientific quality control of papers in OAMJs generally due to the massive volume of papers that they process and publish annually (Erfanmanesh and Teixeira da Silva, 2019[1]; Teixeira da Silva et al., 2019[6]). *Heliyon* was selected because it is an open access (OA) journal, allowing any member of the biomedical community to freely download the papers and independently verify the claims made by Richardson et al. (2024[3]), as well as the findings and observations noted next. Among the 94 *Heliyon* papers in the Richardson et al. (2025[3]) dataset, 23 were flagged for an apparent discrepancy (indicated as “FALSE” in their spreadsheet). The full texts of those 23 papers were examined in detail, manually. Of the 23 papers, published between 2018 and 2022, one was excluded because it was a review (i.e., not original research), only two cases (DOI: 10.1016/j.heliyon.2019.e02756; 10.1016/j.heliyon.2022.e10985) showed a clear discrepancy in both the model and maker in the text (specifically as indicated in the methods section) and in the micrographs in the figures, i.e., a mismatch between textual and figure descriptors of SEM (see Supplementary information, Supplementary Table 1). Cases where there was any doubt, or where only a possible discrepancy was observed in either the model or the maker, were not considered. In the majority of cases (16/23), the methodology did not describe the SEM in detail, or at all, making the papers' methodological premise questionable, and leaving open the option that these authors - most of whom are affiliated with low-income nations - may have used a TPS to complete their SEM analysis.

## Conclusion, Advice, Disclaimer, and Limitations

These cases need to be examined carefully by the journal editors and publisher to determine whether there is validity in any of the provisional claims made in the Richardson et al. (2025[3]) preprint. Of the 23 *Heliyon* papers verified here, two had an unequivocal mismatch in the textual and figure descriptors, while 16/23 had no (or rudimentary) description of the SEM used in the methods, suggesting that all of these papers require some level of correction, fortifying - at least in the studied dataset - the lack of scientific rigor alluded to by Joelving (2024[2]), specifically the quality of peer review at *Heliyon*, which is often offered as a cascade journal option, i.e., authors of papers rejected by other Elsevier journals may be offered to transfer their papers to *Heliyon*. This includes the possibility that a TPS may have been employed, as one potential reason to explain the discrepancy between the textual descriptor and the information in micrographs. The findings of this paper are neither an accusation of ethical misconduct nor fraud, merely an exploratory scientific exercise. This paper only examines one OAMJ, but a paper-by-paper examination of the Richardson et al. (2025[3]) dataset, focusing first on other OAMJs and other OA journals, is warranted. The paper also did not examine other errors or potential issues in the 23 papers, or other techniques, such as transmission electron microscopy.

## Declaration

### Availability of data and material

All data (papers) analyzed can be found in Supplementary information, Supplementary Table 1, via papers' DOIs.

### Conflict of interest

The author has no conflict of interest to declare.

### Author's contributions

Conceptualization, investigation, verification, writing, editing. 

### AI use

AI was not used.

### Disclaimer

A submission to *Heliyon* in March 2025 was rejected on the grounds that the content constituted an expression of concern of a group of the journal's articles that would be investigated accordingly based on the evidence presented. The outcome of that investigation is unknown.

## Supplementary Material

Supplementary information

## Figures and Tables

**Figure 1:**
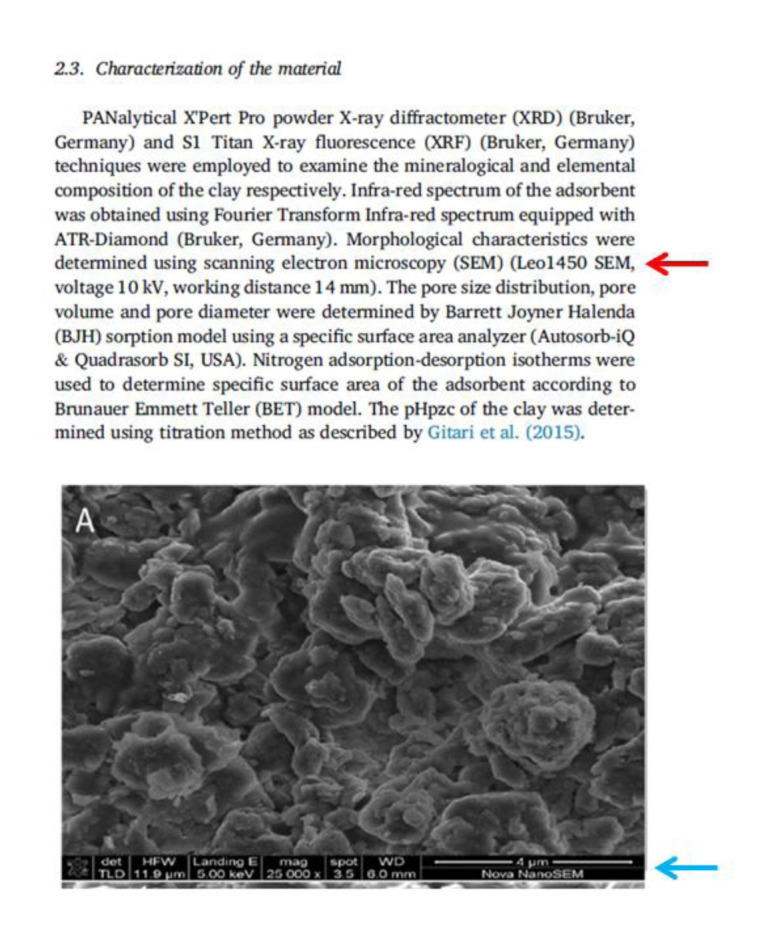
Graphical abstract: Mismatch in SEM descriptors between text and figures of one *Heliyon* paper (DOI: 10.1016/j.heliyon.2019.e02756). The text (top, red arrow) indicates that the SEM used was a Leo1450 (p. 2), but the micrograph in Figure 4 of that paper (p. 5) (bottom, blue arrow) indicates that a Nova NanoSEM was used. Text and figure screenshots reproduced: CC BY-NC-ND license
